# Dimethyl fumarate alleviates allergic asthma by strengthening the Nrf2 signaling pathway in regulatory T cells

**DOI:** 10.3389/fimmu.2024.1375340

**Published:** 2024-04-22

**Authors:** Yanhong Cen, Fangfang Li, Yikui Li, Kaimin Zhang, Farooq Riaz, Kuaile Zhao, Ping Wei, Fan Pan

**Affiliations:** ^1^ Department of Radiation Oncology, Fudan University Shanghai Cancer Center, Shanghai, China; ^2^ Shenzhen Institute of Advanced Technology, Chinese Academy of Sciences, Shenzhen, China; ^3^ Department of Otolaryngology, West China Second University Hospital, Sichuan University, Key Laboratory of Birth Defects and Related Diseases of Women and Children (Sichuan University), Ministry of Education, Chengdu, China; ^4^ Department of Oncology, Shanghai Medical College, Fudan University, Shanghai, China; ^5^ Shanghai Clinical Research Center for Radiation Oncology, Shanghai, China; ^6^ Shanghai Key Laboratory of Radiation Oncology, Shanghai, China

**Keywords:** nuclear factor E2-related factor 2, Nrf2, allergic asthma, dimethyl fumarate, Foxp3, regulatory T Cell

## Abstract

Allergic asthma is a widely prevalent inflammatory condition affecting people across the globe. T cells and their secretory cytokines are central to the pathogenesis of allergic asthma. Here, we have evaluated the anti-inflammatory impact of dimethyl fumarate (DMF) in allergic asthma with more focus on determining its effect on T cell responses in allergic asthma. By utilizing the ovalbumin (OVA)-induced allergic asthma model, we observed that DMF administration reduced the allergic asthma symptoms and IgE levels in the OVA-induced mice model. Histopathological analysis showed that DMF treatment in an OVA-induced animal model eased the inflammation in the nasal and bronchial tissues, with a particular decrease in the infiltration of immune cells. Additionally, RT-qPCR analysis exhibited that treatment of DMF in an OVA-induced model reduced the expression of inflammatory cytokine (IL4, IL13, and IL17) while augmenting anti-inflammatory IL10 and Foxp3 (forkhead box protein 3). Mechanistically, we found that DMF increased the expression of Foxp3 by exacerbating the expression of nuclear factor E2-related factor 2 (Nrf2), and the in-vitro activation of Foxp3+ Tregs leads to an escalated expression of Nrf2. Notably, CD4-specific Nrf2 deletion intensified the allergic asthma symptoms and reduced the in-vitro iTreg differentiation. Meanwhile, DMF failed to exert protective effects on OVA-induced allergic asthma in CD4-specific Nrf2 knock-out mice. Overall, our study illustrates that DMF enhances Nrf2 signaling in T cells to assist the differentiation of Tregs, which could improve the anti-inflammatory immune response in allergic asthma.

## Introduction

Asthma is a chronic respiratory tract disease categorized by variable symptoms, including cough, chest tightness, shortness of breath, and wheezing, with an overall prevalence of 0.9-29% in different countries (1, 2). As one of the most common asthma phenotypes, allergic asthma often arises in juveniles and is linked with sensitization of common aeroallergens, such as those derived from pollen, fungi, cockroaches, house dust mites, and animal dander (3). Exposure to such allergens may disrupt the epithelial barrier, open tight junctions, and induce epithelial cell death (4). It is alarming that despite a high prevalence of allergic asthma, the existing therapies exhibit mild benefits, and other treated patients are highly susceptible to recurrent symptoms (5). Thus, the identification of novel therapeutic drugs is necessary to resolve allergic asthma.

Asthma is not a uniform condition; it encompasses a complex disease that includes various immune mechanisms and diverse clinical manifestations. The underlying allergic asthma pathology begins when alarmins released by damaged epithelial cells induce cytokine secretion by type 2 innate lymphoid cells (ILC2) (6). Moreover, antigen presentation by airway dendritic cells (DCs) participates in differentiating type 2 helper T (Th2) cells. Th2 cells secrete pro-allergic cytokines, including granulocyte-macrophage colony-stimulating factor (GM-CSF), IL3, -4, -5, -9, and -13, which trigger IgE production, mast cells and eosinophilic responses, thereby playing a crucial role in controlling the innate and adaptive immune response to further amplify inflammation (7). Conversely, the production of anti-inflammatory cytokines, for instance, IL10, terminates during the progression of allergic asthma in experimental asthma models and asthmatic individuals (8). Immunosuppressive Foxp3+ regulatory T cells (Tregs) appear to secrete IL10 and negatively regulate inflammation (9). Hence, this suggests that improving the population and function of IL10-secreting Tregs may serve as a promising therapy in patients with allergic asthma.

Dimethyl fumarate (DMF), also identified as Tecfidera, is a fumaric acid ester of fumaric acid accepted by the US FDA and European Medicines Agency to treat relapsing-remitting multiple sclerosis (RRMS) (10–12). DMF treatment ameliorates the disease course of experimental autoimmune encephalomyelitis (EAE) and exerts neuroprotective effects by activating the anti-oxidant nuclear factor erythroid 2 related factor 2 (Nrf2) pathway (13). DMF upregulates Nrf2 level in the nuclear, thus subsequently enhances expression of anti-oxidant genes, such as NAD(P)H quinone oxidoreductase 1 (NQO1), heme oxygenase 1 (HO1), and glutamate-cysteine ligase catalytic subunit (GCLC) in the canonical Nrf2 pathway (13, 14). Current studies have revealed the anti-inflammatory effect of DMF in other disease models. Oral administration of DMF reduces histological lesions and mitigates L-Arginine-induced chronic pancreatitis by protecting pancreatic islet cells (15). Likewise, DMF treatment mitigates dextran sulfate sodium (DSS)-induced colitis. Interestingly, DMF also has shown anti-cancer activity in preclinical models of colon cancer (16), melanoma (17), breast cancer (18), and glioblastomas (12, 19). However, not many studies have explored the effect of DMF on allergic asthma.

Here, we have evaluated the anti-inflammatory consequence of DMF on allergic asthma. Particular attention was paid to understanding how DMF affects the immune microenvironment in asthma and uncovering the mechanism underlying DMF-dependent Treg differentiation in limiting the inflammation, easing the OVA-induced allergic asthma. Our findings exhibited the protective effect of DMF, which enhanced the expression of Nrf2 and led to the differentiation of Tregs, thereby mitigating OVA-induced asthma.

## Results

### DMF relieves OVA-induced allergic asthma.

**Figure 1:**
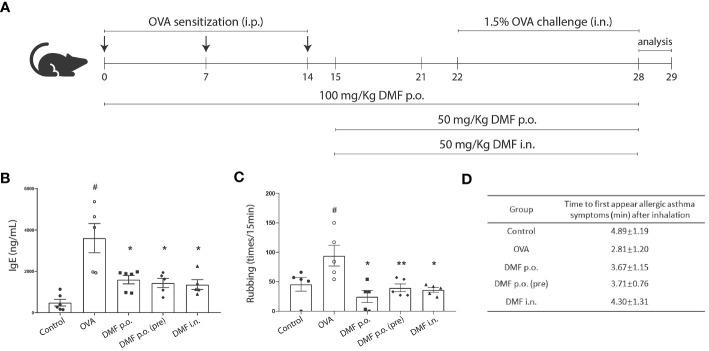
DMF treatment alleviated OVA-induced allergic asthma. **(A)** Schematic illustration showing the protocol to induce allergic asthma model and the DMF treatment used in this study. DMF was administered 100 mg/kg per oral (p.o.) from day 1 to day 28, 50 mg/kg p.o. from day 15 to day 28, or 50 mg/kg intranasal (i.n.) from day 15 to day 28. **(B)** The levels of IgE in the serum from OVA-induced mice with or without DMF treatment. **(C)** The frequency of rubbing was recorded for 5 min after the last OVA intranasal challenge on day 28. **(D)** The allergic symptoms, such as rubbing, sneezing, and abdominal respiration, were monitored for their first appearance in various animal groups. Time in panel D is shown as average ± SD (min). All other data are shown as the mean ± SEM (n = 6 per group). #P < 0.05 versus the control group. **P < 0.01, *P < 0.05 vs OVA group. OVA, Ovalbumin. DMF, dimethyl fumarate.

The role of DMF in alleviating inflammation has been well established (20). However, the effect of DMF in OVA-induced allergic asthma is not well understood. To determine the impact of DMF on clinical respiratory features in OVA-induced allergic asthma, various doses of DMF were given p.o. or i.n. in the OVA-induced allergic asthma model on different days (**Figure 1A**). Given that IgE is a significant contributing component to allergic asthma, we firstly examined the IgE levels in the serum samples. We found that OVA markedly increased the production of IgE in the serum of mice (**Figure 1B**). Meanwhile, DMF treatment significantly reduced the IgE level (**Figure 1B**). Our finding indicates that mice sensitized and challenged with OVA considerably increased the symptoms of rubbing, indicating the hazardous effects of OVA on mice lungs as compared to the control group (**Figure 1C**). However, treatment of DMF in OVA-induced allergic asthma significantly reduced the rubbing frequency as compared to OVA-treated group (**Figure 1C**). This reduced rubbing frequency was more prominent in OVA-induced allergic asthma mice treated with 100mg/kg DMF p.o. for 28 days (**Figure 1B**). In parallel, we also found that the first appearance of clinical features of allergic asthma, including rubbing, sneezing, and abdominal respiration, were appeared earlier in OV-induced mice model. Nevertheless, these clinical features of allergic asthma were delayed in mice administered with DMF, especially in mice administered with 50mg/kg of DMF i.n. (**Figure 1D**). Collectively, these data advocate that the administration of DMF alleviates OVA-induced allergic asthma.

### Treatment of DMF reduces bronchial and nasal inflammation in OVA-induced allergic asthma.

**Figure 2:**
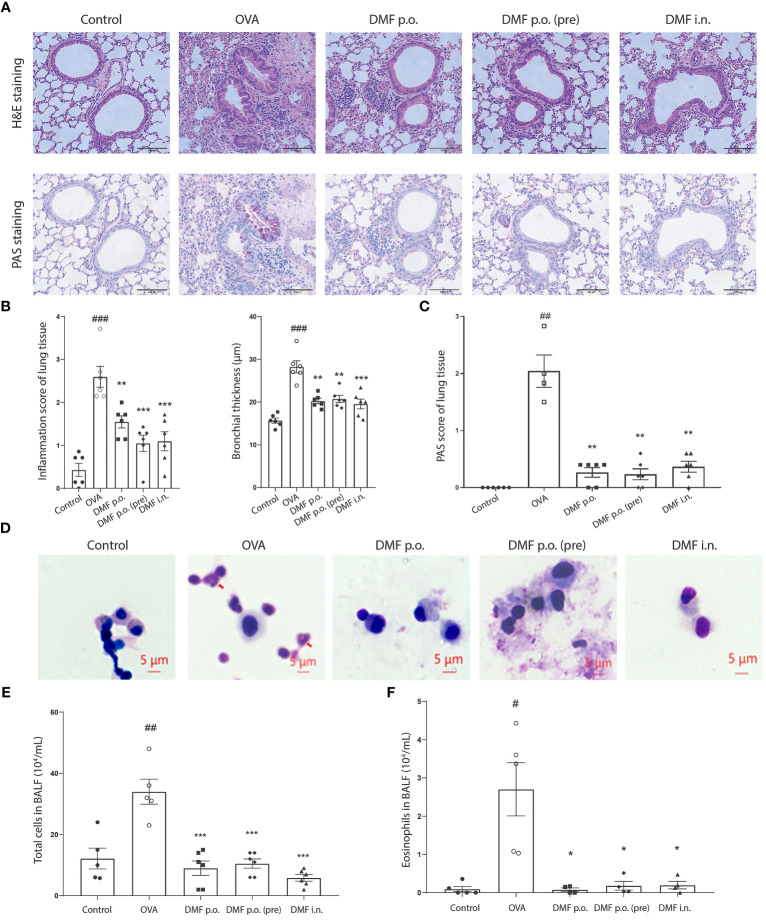
DMF reduced the infiltration of inflammatory cells and inflammation in the lung and BALF of the allergic asthma model. **(A)** H&E and PAS staining was performed to determine the infiltration of inflammatory cells and mucus-secreting goblet cells around the bronchioles, alveoli, and blood vessels observed. Scar bar=100uM. **(B, C)** Bar graphs showing the inflammation score, the bronchial thickness, and the PAS score were calculated from the H&E staining of lung tissues. **(D)** Representative images of BALF cytospin specimens (n = 6 per group). Eos cells were visualized in the OVA or DMF-treated group (red arrow indicated eosinophil). Scar bar=10uM. **(E, F)** The number of totals **(E)** and eosinophil **(F)** cells in each group. Results are shown as the Mean ± SEM (n = 6 per group). ##P < 0.01 versus Naive group. #P < 0.05, ##P < 0.01, ### < 0.001 versus the control group. ***P < 0.001, **P < 0.01, *P < 0.05 vs OVA group. BALF, bronchoalveolar lavage fluid. H & E, hematoxylin-eosin staining. PAS, periodic acid Schiff staining. Eos, eosinophilic granulocyte.

Considering the reduced allergic asthma symptoms in DMF-treated mice, we speculated whether DMF treatment abridged mucus production and airway inflammation in OVA-induced mice. Mice were killed at 29 days, with no significant change in the body weight of control or experimental groups (**Supplementary Figure 1A**). To verify our notion, we collected the lungs and bronchoalveolar lavage fluid (BALF) from mice to examine the impact of DMF on inflammation and the production of mucus in OVA-induced mice. As expected, OVA-induced mice showed increased perivascular and peribronchial inflammation compared to the control group, whereas the DMF administration considerably reduced the OVA-induced peribronchial and perivascular inflammation (**Figures 2A, B**). Similarly, DMF treatment also restricted the OVA-induced mucus hypersecretion in the lung epithelium (**Figures 2A, C**). Moreover, observing the immune cells in BALF cytospin specimens indicates that DMF-treated OVA-induced mice markedly reduced the total number of immune cells compared to OVA-induced mice (**Figures 2D, E**). As infiltration of eosinophils is linked with type 2 inflammatory responses in allergic asthma (21), we counted the number of eosinophils in the BLAF from OVA-induced mice with or without DMF treatment. Interestingly, we found that DMF treatment decreased eosinophils in the BALF of OVA-induced mice (**Figures 2D, F**). This suggests that DMF treatment may mitigate the inflammatory process in OVA-induced asthma.

**Figure 3:**
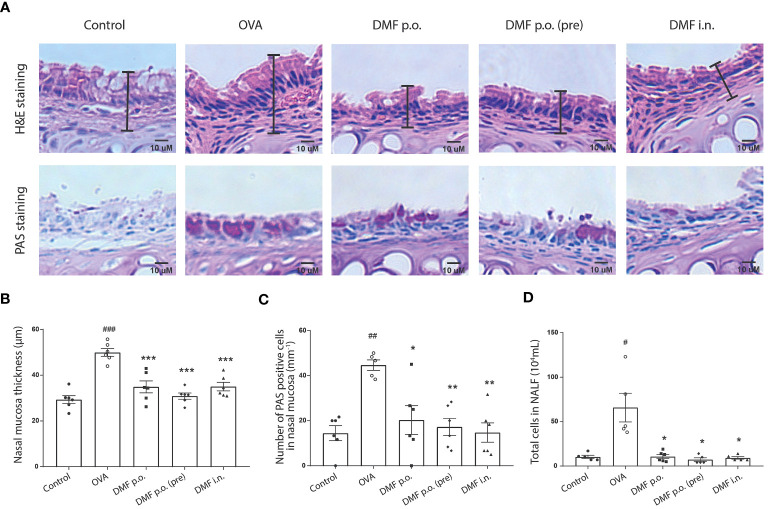
DMF reduced the infiltration of nasal mucus and NALF in the allergic asthma model. **(A)** The histology of nasal mucosa in treatment groups has a less epithelium thickness of nasal mucosa and accumulation of infiltrated inflammatory cells, inflammatory response, and goblet cells. Representative H&E or PAS-stained images of nasal mucosa were shown. Scale bar=10uM. **(B, C)** the bar graphs show the nasal mucosa thickness **(B)** and the PAS-positive cells **(C)** in the nasal mucosa in OVA-induced DMF-treated mice. **(D)** The total cells were calculated from the NALF collected from the *in vivo* experimental groups. Results are shown as the Mean ± SEM (n = 5-6 per group). #P < 0.05, ##P < 0.01, ### < 0.001 versus the control group. ***P < 0.001, **P < 0.01, *P < 0.05 vs OVA group. NALF, nasal lavage fluid.

We have mentioned earlier that DMF treatment decreased the rubbing frequency in mice with OVA-challenged allergic asthma. Thus, we pursued to inspect the impact of DMF treatment on nasal inflammation. Similar to the results of DMF administration on bronchial inflammation in OVA-induced mice, the histological analysis of nose tissue revealed that administration of DMF in OVA-induced mice considerably reduced the inflammatory cell accumulation and the nasal thickness (**Figures 3A, B**). Besides, DMF treatment also affects the number of mucus-secreting cells in OVA-induced allergic asthma (**Figures 3A, C**). Strikingly, DMF-treated OVA-induced mice reduced the accretion of immune cells in nasal lavage fluid (NALF) (**Figure 3D**). Collectively, our data represent the protective role of DMF in the OVA-induced allergic asthma model.

### Administration of DMF eases inflammation in OVA-induced mice by Foxp3/Treg

**Figure 4:**
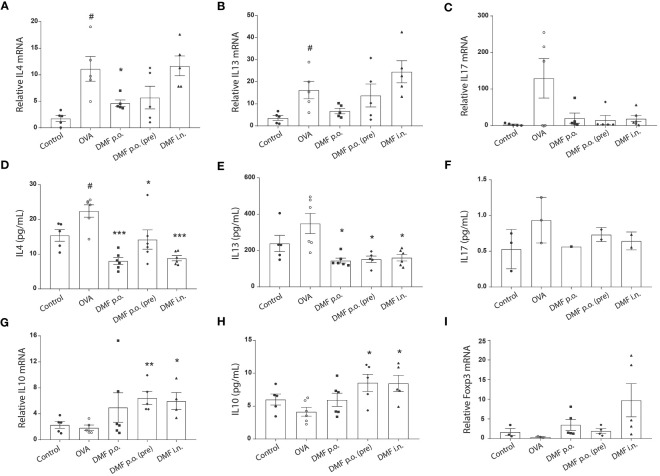
DMF reduces the inflammatory markers in the lungs of the allergic asthma model. **(A–C)** RT-qPCR was performed to analyze the mRNA expression of Th2 inflammatory cytokines IL4 **(A)** and IL13 **(B)** and Th17 cytokine IL17 **(C)** in the lungs of OVA-induced mice with or without DMF treatment. **(D–F)** ELISA was performed to evaluate the protein expression of Th2 inflammatory cytokines IL4 **(D)** and IL13 **(E)** and Th17 cytokine IL17 **(F)** in the lungs of OVA-induced mice with or without DMF treatment. **(G)** RT-qPCR was performed to determine the mRNA expression of anti-inflammatory cytokine IL10 in the lungs of OVA-induced mice with or without DMF treatment. **(H)** ELISA was performed to determine the protein expression of anti-inflammatory cytokine IL10 in the lungs of OVA-induced mice with or without DMF treatment. **(I)** RT-qPCR was performed to determine the mRNA expression of Foxp3 in the lungs of OVA-induced mice with or without DMF treatment. All other data are shown as the mean ± SEM (n = 6 per group). #P < 0.05 versus the control group. ***P < 0.001, **P < 0.01, *P < 0.05 vs OVA group. OVA, Ovalbumin. DMF, dimethyl fumarate.

Both Th2 and Th17 are the primary inflammatory cells involved in the progression of allergic asthma (22, 23). Meanwhile, Tregs are anti-inflammatory cells that may suppress the activation and function of Th2 and Th17 inflammatory cells (24, 25). Since we observed reduced nasal and bronchial inflammation in the DMF-treated OVA-challenged allergic model, we aimed to investigate the expression of cytokines secreted from Th2 and Th17. RT-qPCR analysis exhibited that OVA treatment significantly increased the mRNA level of inflammatory IL4 and IL13, whereas no significant difference was observed in the mRNA level of IL17. Despite a slight decrease, DMF treatment failed to significantly alter the mRNA expression of these inflammatory cytokines (**Figures 4A–C**). Nevertheless, at the protein level, we observed that DMF treatment considerably reduced the levels of inflammatory cytokines IL4 and IL13 but not IL17(**Figures 4D–F**). Conversely, evaluation of anti-inflammatory genes, such as IL10, showed that DMF treatment in OVA-induced mice led to an apparent upsurge in the level of IL10 at both mRNA and protein level compared to OVA-induced mice (**Figures 4G–H**). Meanwhile, we also observed a slight increase in the expression of Foxp3 mRNA in lung tissues of OVA-induced DMF-treated mice but it was not significant (**Figure 4I**) These findings indicate that retarded bronchial and nasal inflammation is highly associated with the improved increased Treg population.

### DMF modulates Nrf2 signaling to promote Treg

**Figure 5:**
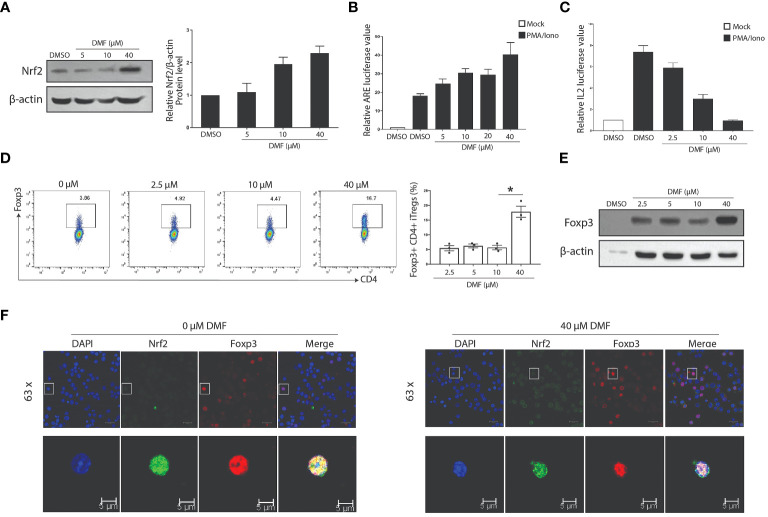
DMF influences the expression and function of Nrf2 and Foxp3. **(A)** Western blot analysis shows that the treatment of DMF promotes the expression of Nrf2 in a dose-dependent manner. **(B)** Luciferase assay was performed to determine the effect of DMF on the activation of Nrf2/ARE pathway. **(C)** The luciferase activity evaluated the effect of DMF on the IL-2 activity. Jurkat T cells were incubated overnight after electro-transfection, and were treated with DMF in 0, 2.5, 10, and 40uM for seven h, stimulated without or with 40nM PMA and 1uM ionomycin for 6 h. DMF suppresses the secretion of IL2. **(D, E)** Naive CD4+ T cells were isolated from the lymphoid and spleen tissue of WT C57BL/6 mice and activated under Treg polarization conditions using 2ug/mL anti-CD3, 1ug/mL anti-CD28, 10ng/mL IL2 and 2.5ng/mL TGF-β. Cells were harvested at 48h. FACS **(D)** and western blot **(E)** assessed the protein levels of Foxp3. **(F)** Niave T cells polarized under Treg skewing conditions were processed to determine the co-localization of Foxp3 and Nrf2 in the absence or presence of DMF. All experiments were repeated independently at least three times. *P < 0.05. Data is shown as the Mean ± SEM.

Activation of Nrf2 limits allergen-induced asthma (26), whereas DMF promotes the expression of Nrf2 to modulate inflammation (27). Following this, we hypothesized that the DMF-induced increased Tregs population depends on Nrf2 signaling. To verify our hypothesis, we first used western blot analysis to estimate the impact of DMF on the Nrf2 level. As anticipated, treatment of DMF in Jurkat T cells heightened the Nrf2 expression in a dose-dependent manner (**Figure 5A**). Likewise, DMF treatment also strengthened anti-oxidant response element (ARE)-regulated transcription activity in HEK293T cells in a dose-dependent manner (**Figure 5B**), thus suggesting that DMF-dependent upregulation of Nrf2 improves the anti-oxidant response in cells.

To evaluate the effect of DMF on Tregs, we first ascertained the activity of IL2 secretion by transfecting IL2 reporter plasmid in Jurkat T cells induced with PMA/Iono. Data indicate that DMF suppresses the activity of IL2 (**Figure 5C**). Consequently, naive CD4+ T cells isolated C57BL/6 WT mice were induced under Treg skewing condition using 1µg/mL anti-CD28, 2µg/mL anti-CD3 and 2.5ng/mL TGF-β for 48 hours, and treated with various doses of DMF to validate the effect of DMF on Treg differentiation. Data indicate that a higher dose of DMF (40µmol) considerably increased the Foxp3+ iTreg compared to iTregs without DMF (**Figure 5D**). Correspondingly, western blot analysis showed a similar trend, i.e., increased Foxp3 expression in iTregs treated with 40µmol of DMF (**Figure 5E**). Immunofluorescence assay shows that DMF (at the dose of 40µmol) increased the expression and nuclear localization of Foxp3 compared to iTregs deprived of DMF (**Figure 5F**). These data suggest that DMF activates the Nrf2 signaling to induce Tregs.

### Genetic deletion of Nrf2 mitigates Treg differentiation

**Figure 6:**
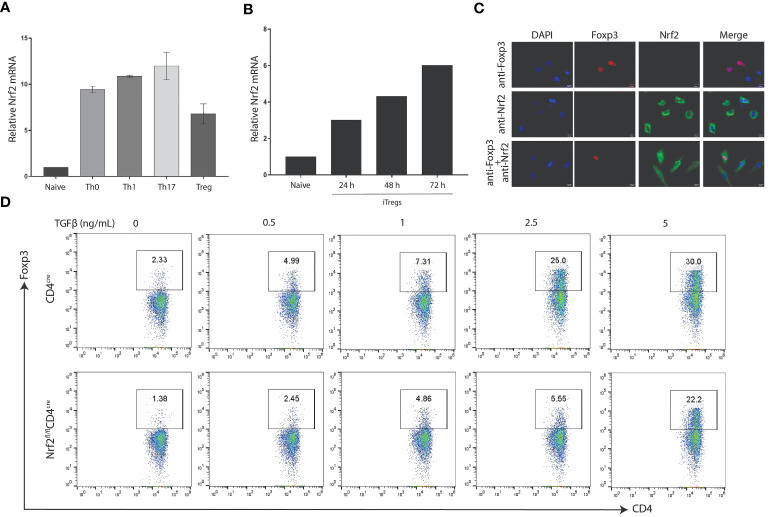
Nrf2 is required for Foxp3 differentiation. **(A)** Naive CD4+ T cells were isolated from the spleen and lymph nodes of WT C57BL/6 mice and activated under polarizing conditions to generate the indicated T-helper subset. The RT-qPCR was performed to determine the expression of Nrf2 mRNA. **(B)** Naïve CD4+ T cells induced under Treg polarization conditions were processed to validate the expression of Nrf2 mRNA by utilizing RT-qPCR. **(C)** Hela cells were transfected with Foxp3 or NFE2L2 plasmids. Representative confocal microscopy images were taken 48 hours after transfection, visualizing for Foxp3 (red), Nrf2 (green), and DAIP (blue). Shown are representative of three different biological replicate experiments. **(D)** Nrf2-deficient CD4 cells were isolated from the WT or Nrf2^fl/fl^CD4^cre^ mice and induced under iTreg conditions to validate the difference in the Treg population between the two groups. Data is shown as the Mean ± SEM.

To understand the function of Nrf2 in the differentiation of CD4+ T cell subsets, we induced naïve CD4+ T cells collected from the lymph nodes and spleen of WT mice and induced under different CD4-skewing conditions. Intriguingly, RT-qPCR analysis advocates that Nrf2 is highly expressed in different subsets of CD4+ T cells, such as Th0, Th1, Th17 and Treg subsets (**Figure 6A**). Validation of Nrf2 expression in iTregs at different time intervals illustrate that Nrf2 levels in iTregs elevate in a time-dependent manner (**Figure 6B**). Since we observed that DMF, an activator of Nrf2, remarkably enhanced Foxp3 expression, we aimed to validate whether DMF-dependent increased Foxp3 was dependent on the level of Nrf2 in T cells and that Nrf2 participates in the iTreg differentiation. Firstly, we transfected Foxp3 and Nrf2 overexpressing plasmids in HeLa cells and utilized an immunofluorescence assay to detect the localization of Nrf2 and Foxp3. Confocal microscopic results show that Nrf2 was predominantly expressed in the cytoplasm of the HeLa cells, whereas Foxp3 was localized in the nucleus of the HeLa cells. However, co-expression of Nrf2 and Foxp3 showed that Foxp3 was localized in the nucleus, while Nrf2 was localized throughout the cells, including the nucleus, in the dual positive cells (**Figure 6C**). This suggests that Nrf2 actively participates in the differentiation of Foxp3+ Tregs.

These findings demonstrate the critical role of Nrf2 in Treg development. Finally, we generated mice with Nrf2 deletion in their CD4+ T cells (Nrf2^fl/fl^CD4^cre^). Naïve CD4+ T cells were collected from Nrf2^fl/fl^CD4^cre^ and CD4^cre^ and induced under Treg polarization conditions using different doses of TGFβ. Afterwards, flow cytometry was performed to compare the Treg development between the two groups. Strikingly, outcomes indicate that CD4-specific deletion of Nrf2 distinctly reduced the differentiation of TGFβ-induced Tregs than that of the control group (**Figure 6D**). Together, it was concluded that elevated expression of Nrf2 is necessary for an improved Treg-dependent anti-inflammatory immune response in treating allergic asthma.

### DMF effectively alleviated the inflammation response in the lungs of asthma mice.

**Figure 7:**
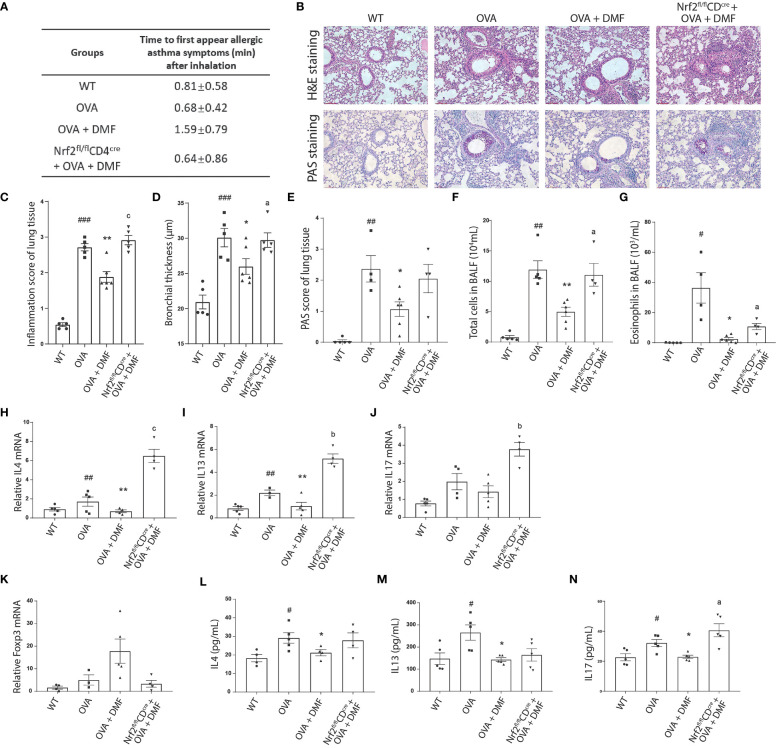
Oral administration of DMF alleviates the inflammatory response in the lungs of asthmatic mice. **(A)** The allergic symptoms, such as rubbing, sneezing, and abdominal respiration, were monitored for their first appearance in OVA-induced WT or Nrf2^fl/fl^CD4^cre^ mice after p.o. administration of DMF. **(B–E)** Histological analysis was performed on the lung tissue collected from the OVA-induced WT or Nrf2^fl/fl^CD4^cre^ mice after p.o. DMF treatment using H&E and PAS staining. Inflammation score, bronchial thickness, and PAS staining score were calculated. **(F, G)** After DMF treatment, the total number of immune cells and eosinophils was calculated through the microscopic examination of BALF collected from the OVA-induced WT or Nrf2^fl/fl^CD4^cre^ mice. **(H, I)** RT-qPCR was performed to examine the expression of Th2 cytokines IL4 **(H)**, IL13 **(I)**, and Th17 cytokine IL17 **(J)** in the lung of OVA-induced WT or Nrf2^fl/fl^CD4^cre^ mice in the presence or absence of DMF. **(K)** RT-qPCR was utilized to study the Treg transcription factor Foxp3 **(K)** level in the lung of OVA-induced WT or Nrf2^fl/fl^CD4^cre^ mice in the presence or absence of DMF. **(L–N)** ELISA was conducted to inspect the level of circulatory Th2 cytokines IL4 **(L)**, IL13 **(M)**, and Th17 cytokine IL17 **(N)** in the lungs of OVA-induced WT or Nrf2^fl/fl^CD4^cre^ mice in the presence or absence of DMF. Time in **(A)** is shown as average ± SD (min). All other data are shown as the mean ± SEM (n = 6 per group). #P < 0.05, ##P < 0.01, ###P < 0.001 versus the WT group. ***P < 0.001, **P < 0.01, *P < 0.05 vs OVA group or OVA+DMF group. P^a^ < 0.05, P^b^ < 0.01 and P^c^ < 0.001.

As we hypothesized that DMF strengthens the population of Tregs by upregulating Nrf2, which plays a protective role in allergic asthma, we were subjected to exploring the CD4-specific role of Nrf2 in allergic asthma and immune response. For this, we induced Nrf2^fl/fl^CD4^cre^ under OVA-induced allergic asthma conditions in the presence of DMF (p.o.). The appearance of disease symptoms was calculated as previously. Our data displayed that p.o. administration of DMF considerably delayed the onset of disease symptoms in OVA-induced WT mice; nevertheless, DMF failed to exert its protective effect in OVA-induced Nrf2^fl/fl^CD4^cre^ mice (**Figure 7A**). Meanwhile, no considerable body change was observed among these experimental groups (**Supplementary Figure 1B**). Histological analysis of lung tissue collected from the DMF treated OVA-induced WT or Nrf2^fl/fl^CD4^cre^ mice showed that although DMF reduced the inflammation and bronchial thickness in the OVA-induced WT mice, it failed to induce significant change in these histological parameters in the Nrf2^fl/fl^CD4^cre^ mice (**Figures 7B–D**). Consistently, no change in the PAS-positive cells (**Figures 7B, E**) and the total number of leukocytes or eosinophils was detected in the DMF-treated OVA-induced Nrf2^fl/fl^CD4^cre^ mice (**Figures 7F, G**). Similar to our previous data, DMF noticeably mitigated the expression of inflammatory cytokines IL4, IL13, and IL17 in OVA-induced WT mice (**Figures 7H–J, L–N**). In contrast, deletion of Nrf2 in CD4 cells leads to a considerable increase in these inflammatory genes (**Figures 7H–J, L–N**). This illustrates that DMF exerts protective effects in allergic asthma via upregulation of Nrf2, whereas CD4-specific deletion of Nrf2 exacerbates the inflammatory response in allergic asthma.

Accordingly, DMF treatment in iTregs decreased Foxp3 expression and Treg differentiation *in vitro*. Therefore, we speculated the impact of DMF on the level of anti-inflammatory Foxp3 in the WT or Nrf2^fl/fl^CD4^cre^ mice after inducing OVA-allergic asthma. We found that the administration of DMF intensified the mRNA level of Foxp3 in the lungs of the OVA-induced animal model, whereas Nrf2^fl/fl^CD4^cre^ mice exhibited that DMF treatment failed to upregulate the expression of Foxp3 (**Figure 7K**). Instead, it led to nearly diminished expression of Foxp3, suggesting that DMF-dependent increased Treg differentiation and Foxp3 expression was dependent on the Nrf2 in CD4+ T cells.

## Discussion

Allergic asthma is a complex inflammatory condition often accompanied by sophisticated immune responses. Current research has demonstrated that the pathophysiology of asthma is directly connected to the disparity of T cell subsets and associated cytokines (28). Previously, it was indicated that DMF has a significant anti-inflammatory effect in various diseases; however, its therapeutic potential in allergic asthma has not been examined. Thus, our current study evaluated the efficacy of DMF treatment and its impact on infiltrating immune cells in an OVA-triggered allergic mouse model. Our results demonstrate that DMF reduced the infiltration of inflammatory cells in the nasal and bronchoalveolar tissues. This reduced inflammatory potential in DMF-treated OVA-induced mice was due to the increased population of immune suppressive Tregs with increased expression of their anti-inflammatory cytokines. Mechanistically, we established that DMF activates the Nrf2 pathway in Treg cells to boost the expression of Foxp3, thereby enhancing the population of Tregs. This was further validated in CD4-specific Nrf2 knock-out mice, which exhibited a deteriorated population of TGFβ induced Tregs.

In allergic asthma, oxidative stress contributes to the overproduction of mucus, airway inflammation and remodeling, augmented secretion of pro-inflammatory cytokines, and hyperresponsiveness (29, 30). Systematically, as allergic asthma progresses, the production of pro-inflammatory cytokines subsidizes the infiltration of eosinophils in the lung tissue (31). Recruitment of eosinophils further activates the Th2-type allergic immune responses by secreting IL13 (32, 33), consequently exacerbating allergic asthma (34). Thus, the regulation of pro-inflammatory cytokines is vital in restricting asthma and reducing eosinophil infiltration, and pro-inflammatory genes are prognostic indicators of allergies and asthma (35, 36). Inhibition of type 2 inflammation to treat allergic asthma has shown effects in clinical trials (37). Omalizumab, a monoclonal antibody targeting IgE, can restrain airway responses to inhaled allergens (38), improve asthma control and reduce exacerbation rate (39). Antibodies targeting IL5 (Mepolizumab) or IL5 receptor α-chain (Benralizumab) reduce the asthma exacerbation rate and the daily dose of oral corticosteroids (40). On the other hand, substantial evidence indicates that allergen-specific immunotherapy (AIT) inducing immunological tolerance in allergic asthma patients can reduce short-term symptoms and medication scores (41). Considering the restrictions of prevailing therapies, we have identified the novel feature of DMF in combating allergic asthma by reducing the production of pro-inflammatory cytokines, i.e., IL4, IL13, and IL17, in the bronchoalveolar tissue. We also noticed that DMF administration markedly mitigated the infiltration of eosinophils in the lung tissue. However, DMF treatment in T cell-specific Nrf2 KO mice failed to make some differences in number of eosinophils and the protein expression levels of IL-4 and IL-13. This apparent lack of effect on circulating IL-4, IL-13, and eosinophil levels in Nrf2^fl/fl^CD4^cre^ mice suggests a complex interplay between different immune response cells, where Nrf2 plays a critical role and it needs further investigations.

On the contrary, impairment of Treg function is linked with the severity of asthma in humans as well as in mice models (42, 43). Treg cells can hinder the activity of an array of effector T cells and are crucial for sustaining immunological tolerance and homeostasis (44). An elevated Treg population is estimated to be a good prognostic indicator as it helps maintain the Th2/Treg and Th17/Treg balance in the bronchoalveolar tissue to reduce the symptoms of allergic asthma (45, 46). Treg cells inhibit immunological inflammation in asthmatic patients’ airways primarily via direct cell-to-cell contacts and the release of the cytokine IL10, which aids in immunosuppression. IL10 significantly inhibits inflammatory cytokine production and inflammatory cell growth (47, 48). Our study adds that DMF reduces inflammation in OVA-induced allergic asthma through increased production of IL10 along with an elevated population of Tregs, which may lead to a diminished population of infiltrating pro-inflammatory cells in the lung tissue and BALF.

Activation of Nrf2 is predominantly involved in the anti-oxidant pathway. It is known that the nuclear translocation of Nrf2 is pivotal to responding to chemical or oxidative stress through its interaction with ARE (49). Ample investigations have shown that genetic ablation of Nrf2 worsens the inflammation and increases the oxidative stress in numerous disease models, including pulmonary fibrosis (50), sepsis (51), acute lung injury (52), COPD exacerbations (53), and emphysema (54). Pharmacological or genetic activation of Nrf2 decreases oxidative stress and inflammation (55). The role of DMF in activating Nrf2 has been well-established in dendritic cells (56), renal tubular epithelial cells (57), and tumor-associated macrophages (18). However, our study is engrossed in clarifying the anti-inflammatory impact of DMF in allergic asthma through Nrf2 signaling. We determined that DMF reduces airway inflammation, hypersecretion of mucus, and oxidative damage by promoting Nrf2 signaling. This was in accordance with a recent study in which authors demonstrated that Nrf2 deficiency, largely in the germline, showed abrogated Foxp3 (58), and the mice with Nrf2 deficiency are prone to the development of autoimmune diseases (59). Despite the striking potential of DMF in alleviating allergic asthma and enhancing the differentiation of Tregs through Nrf2, our data presents a limited landscape of role of DMF in Nrf2-dependent increased Tregs in allergic asthma. Therefore, we urge that further studies are needed to fully characterize the role of Nrf2 in T cell development and shaping the intrapulmonary immune landscape, mainly in the context of allergic asthma.

Although numerous studies have urged that an increased Nrf2 and an augmented and functional Treg population is necessary to treat allergic asthma, our study notably demonstrates that DMF treatment surges the Nrf2 expression predominately in the Treg, where Nrf2 acts as a helping hand in promoting the level of Foxp3 and the differentiation of Tregs. Moreover, the depletion of Nrf2 in T cells reduces the population of Foxp3+ Tregs, suggesting the critical role of Nrf2 in Treg development. Overall, our study concluded that DMF treatment could efficiently suppress the inflammatory response in allergic asthma by improving the Treg population by activating the Nrf2 signaling pathway.

## Materials and methods

### Animals

Female Balb/c mice, 6 to 8-week-old, were used as experiment animals and purchased from Guang Dong Vital River Laboratory Animal Technology Co., Ltd (Guangdong, China). C57BL/6 background mice were backcrossed with Nfe2l2-loxP/loxP mice to acquire Nrf2^fl/fl^ mice. Nrf2^fl/fl^ mice were crossed to CD4^cre^ mice to generate mice with CD4 cell-specific Nrf2 knock-out. Mice were accustomed to the laboratory housing for 3 to 4 days and housed in a specific pathogen-free condition, with a temperature of 20-26°C, a relative humidity of 40-70% and an alternate time of day and night at 12h. Mice were housed with ad libitum access to water and diet. The Institutional Animal Care and Use Committee of the Shenzhen Institute of Advanced Technology has granted the ethical approval of experimental animals.

### Model establishment and DMF administration

The Balb/c Mice were casually divided into five groups (n=5-6): (1) WT group, (2) OVA group, (3) DMF peros (p.o) group, (4) DMF p.o prevention (pre) group and (5) DMF intranasal (*i.n*). The OVA-induced allergic asthma animals were immunized by intraperitoneal injection of 200 µL phosphate‐buffered saline (PBS), including 80 μg OVA (Grade V, Sigma, USA) and 2 mg of Alum Adjuvant (Sigma, USA) to effectively stimulate the immune response on days 0, 7, 14. The Naive group was left treated with vehicle (PBS). Mice in DMF *p.o* and DMF *p.o* pre groups received treatment once a day with 100 mg/kg DMF by oral administration for 14 or 28 days, respectively. The DMF i.n group was treated with 50 mg/kg DMF by intranasal daily from days 15 to days 28. Next, mice were challenged via intranasal administration of 120 mg OVA (1.5% in PBS) daily for 7 days. Mice were euthanized after the last OVA challenge 24h. The experimental program is displayed in **Figure 1A**.

To study the effect of DMF and Nrf2 on Treg development, we generated Nrf2^fl/fl^CD4^cre^ on C57BL/6 background. The rationale for prioritizing the C57BL/6 mouse strain over the Balb/c mouse strain for T cell biology is that a previous study showed that naïve CD4+CD25− cells from C57BL/6 mouse strain are more prone to becoming Tregs (60). To validate the efficacy of DMF in Nrf2^fl/fl^CD4^cre^ mice, DMF was administered p.o.

### Allergic asthma symptom symptoms

To observe the phenotypic changes in our animal model, we recorded the time to the first appearance of any of the asthma symptoms, including rubbing, sneezing, and abdominal respiration. Alongside that, the number of nasal rubbing times for 15 minutes was counted to estimate the early response of the allergy after the last OVA challenge.

### Collection and Analysis of BALF and NALF

The left lung and the anterior naris were lavaged via the tracheal tube with phosphate buffer saline to collect Bronchoalveolar lavage fluid (BALF) and Nasal lavage fluid (NALF), centrifuged at 6,000rpm for 15min at 4°C. The supernatant was collected to perform an enzyme-linked immunosorbent assay (ELISA) to quantify cytokine release. Cytokine ELISA kits were used, including IL4, IL13, IL10 and IL17 (elabscience). Cytokine release was detected and analyzed as per the manufacturer’s instructions. The BALF and NALF cells were resuspended with cold PBS. The cell numbers were counted by using a hemocytometer. The left cells were centrifuged onto cytospin slides, stained with Giemsa and differentially counting under light microscopy.

### Histopathological image analysis

Mice lungs and nasals were placed in 4% paraformaldehyde (PFA) fix solution for 3 days at 4°C. Then, the samples were embedded in paraffin blocks. The nasal tissues were decalcified at room temperature for 2-3 days. The tissues were sectioned at 4um thickness. Hematoxylin and eosin (H&E) staining was performed to estimate general morphology and inflammatory cell infiltration. The lung tissue HE staining scoring standards is defined as described by Myou et al. (61). Periodic acid-Schiff (PAS) staining was used to estimate the accumulation of mucus-containing cells in tissue. The method described by Padrid was used to quantify goblet cell hyperplasia (62).

### Cell culture and transfection

HEK293 and Hela cells were grown in DMEM + 10% FBS media. Hela and HEK293 were transfected using a transfection reagent suggested by the manufacturers. Jurkat T cells from human origin were cultured in RPMI 1640 + 10% FBS media. The plasmids were transfected into Jurkat cells by electroporation.

### Quantitative real-time PCR

Total RNA was isolated from the lungs and mouse primary cells utilizing the TRIzol (Invitrogen) technique. Subsequently, RNA was reverse-transcribed into cDNA using a One-Step gDNA Removal and cDNA Synthesis SuperMix kit (TransGen Biotech) according to the instructions provided by the maker. Quantitative real-time PCR (SYBR green) was used to compare gene expression levels to those of β-actin. The primer sequences utilized are as follows (**Table 1**):

**Table 1:** List of primers used in this study.

Gene	Forward primer	Reverse primer
IL4	CCCCAGCTAGTTGT CATCCTG	CAAGTGATTTTTGTCG CATCCG
IL13	CAGCCTCCCCGAT ACCAAAAT	GCGAAACAGTTGCTT TGTGTAG
IL10	TACCTGGTAGAAG TGATGCC	TAGACACCTTGGT CTTGGAG
Foxp3	CCCATCCCCAGGA GTCTTG	ACCATGACTAGG GGCACTGTA
IL17	CAGACTACCTCAACCG TTCCAC	TCCAGCTTTCCCT CCGCATTGA
β-actin	CATTGCTGACAGG ATGCAGAAGG	TGCTGGAAGGTGGA CAGTGAGG

### *In vitro* Treg differentiation

Freshly naive CD4^+^ T cells were collected from the lymphoid and spleen tissue of wild-type C57BL/6 mice by using a commercial kit for naïve CD4^+^ T cell isolation (Miltenyi). For *in vitro* activation, 1×10^6^ cells were seeded in 24-well plates, activating with 2 µg/mL anti-CD3 (invivoMAb) and 1 µg/mL anti-CD28 (invivoMAb) in the presence of 10 ng/mL IL2 (R&D) and 2.5 ng/mL TGF-β (R&D) for 24, 48 or 72h.

### Flow cytometry

Cells were spun in a centrifuge at 1,400rpm for 5 minutes, followed by staining with live/dead dye (Thermo Fisher) and CD4 eFluor450 mAb (eBioscience) dissolved in PBS at 1:1000 for 1×10^6^ cells for 15 minutes at the ambient temperature in dark conditions. Cells were rinsed and reconstituted in a BD Cytofix/Cytoperm fixation/permeabilization kit (BD). Cells were fixed and permeabilized for 45 minutes at 4°C in the dark. After washing with permeabilization buffer, lymphocytes were then stained with Foxp3 PE mAb (eBioscience) for 45 minutes at 4°C. Cells were subsequently rinsed with permeabilization buffer and reconstituted in FACS buffer to set up for flow cytometry.

### Immunofluorescence assay

In-vitro iTregs generated from C57BL/6 mice were spun at 1,400 rpm for 5 minutes to attach to poly-L-lysine coated slides for the microscopic examination. Cells were fixed with 4% PFA for 15 minutes at ambient temperature, washed three times with 1×PBS, blocked with 10% BSA for 1 hour, permeabilized with 0.5% Triton X-100, and incubated with antibodies to Nrf2 (CST, 1:300) and Foxp3 (Invitrogen, 1:400) overnight at 4°C. The following day, the cells were rinsed three times with 1×PBS and treated for one hour with anti-rabbit-488 and anti-mouse-555 (Invitrogen). The nuclei of cells were stained with DAPI dye (1:500). The pictures were captured using a laser confocal microscope (LEICA).

### Statistical analysis

All data from the research we conducted were examined using GraphPad Prism 8.0. Student’s t-test was implemented to perform the statistical analysis of the data. All measurements are reported as mean ± standard error (SE). P-values < 0.05 were used to designate statistical significance. * P < 0.05, ** P < 0.01; *** P < 0.001.

## Data availability statement

The original contributions presented in the study are included in the article/**Supplementary Material**. Further inquiries can be directed to the corresponding author.

## Ethics statement

The animal study was approved by the Institutional Animal Care and Use Committee of the Shenzhen Institute of Advanced Technology. The study was conducted in accordance with the local legislation and institutional requirements.

## Author contributions

YC: Investigation, Validation, Writing – review & editing. FL: Formal analysis, Validation, Writing – review & editing. YL: Methodology, Validation, Writing – review & editing. KaZ: Formal analysis, Validation, Writing – review & editing. FR: Formal analysis, Writing – review & editing. KuZ: Conceptualization, Formal analysis, Funding acquisition, Investigation, Supervision, Writing – review & editing. PW: Conceptualization, Funding acquisition, Investigation, Methodology, Supervision, Writing – review & editing. FP: Conceptualization, Formal analysis, Funding acquisition, Supervision, Writing – original draft, Writing – review & editing.
