# Supplementary material for: Dimethyl fumarate alleviates allergic asthma by strengthening the Nrf2 signaling pathway in regulatory T cells
Source: Front Immunol. 2024 Apr 22;15:1375340. doi: 10.3389/fimmu.2024.1375340 (PMC11070462; doi:10.3389/fimmu.2024.1375340)
Supplement: Supplementary file 1 [file DataSheet_1.docx]

**Dimethyl fumarate alleviates allergic asthma by strengthening the Nrf2 signaling pathway in regulatory T cells**

Yanhong Cen^1,2,4,5,6^, Fangfang Li^2,3^, Yikui Li^2^, Kaimin Zhang^2^, Farooq Riaz^2^, Kuaile Zhao^1,4,5,6*^, Ping Wei ^2,3*^, Fan Pan^2*^

1 Department of Radiation Oncology, Fudan University Shanghai Cancer Center, Shanghai 200032, China
2 Shenzhen Institute of Advanced Technology, Chinese Academy of Sciences (CAS), Shenzhen, China.

3 Department of Otolaryngology, Children's Hospital of Chongqing Medical University, National Clinical Research Center for Child Health and Disorders, Ministry of Education Key Laboratory of Child Development and Disorders, Chongqing Key Laboratory of Translational Medical Research in Cognitive Development and Learning and Memory Disorders, Chongqing, China.

4 Department of Oncology, Shanghai Medical College, Fudan University, Shanghai 200032, China

5 Shanghai Clinical Research Center for Radiation Oncology, Shanghai 200032, China

6 Shanghai Key Laboratory of Radiation Oncology, Shanghai 200032, China

**Supplementary Figures:**


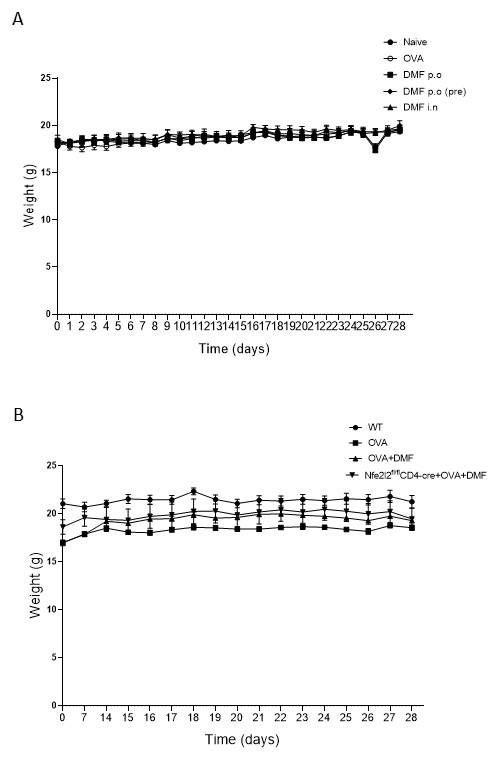


**Suppl. Fig. 1: DMF didn’t change body weight in the allergic asthma.** (A) administration of DMF via various routes in the OVA-induced allergic asthma model failed to induce body weight changes. (B) DMF treatment in the OVA-induced WT or Nrf2^fl/fl^CD4^cre^ mice didn’t lead to a significant change in the body weight.


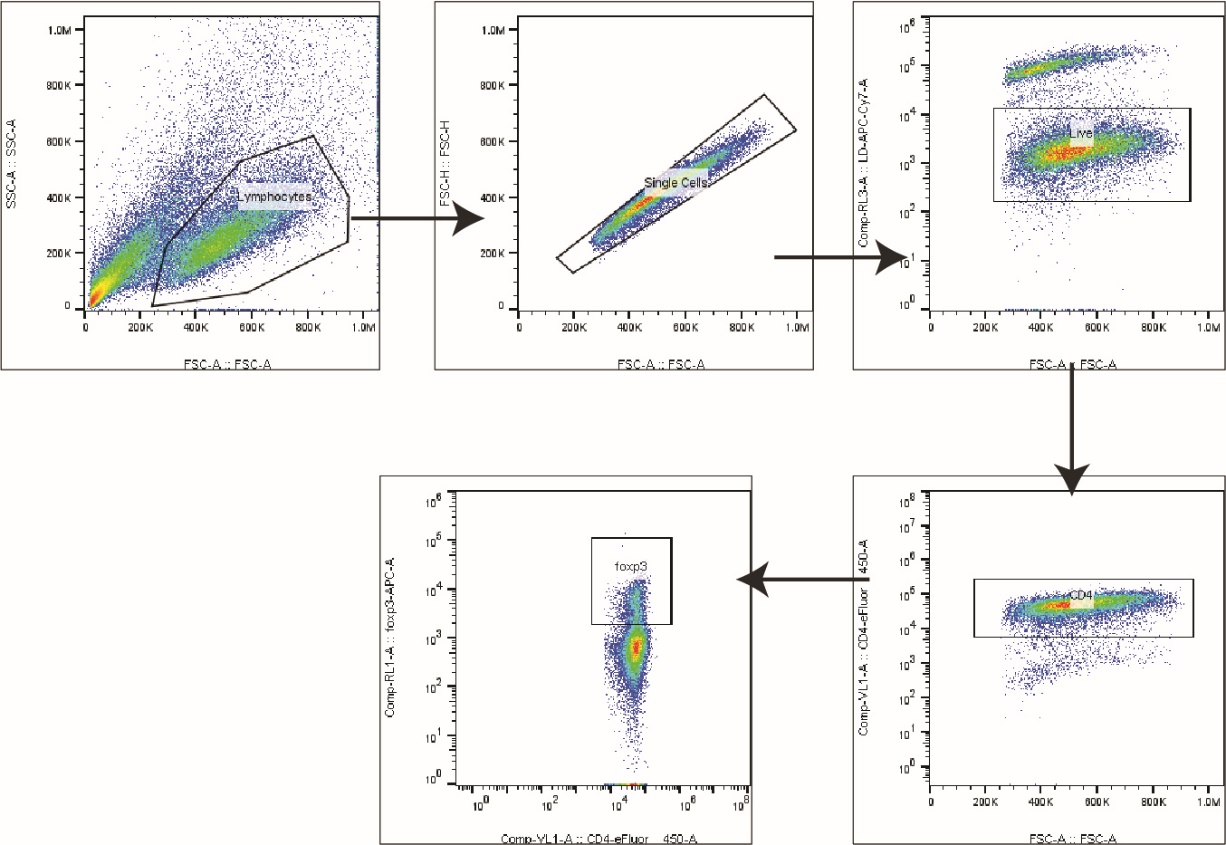


**Suppl. Fig. 2: Gating strategy for flow cytometry analysis used in this study.** A gating strategy was used to define the population of foxp3+ T cell subsets from Naive CD4+ T cells after induction.
