# Supplementary material for: Dimethyl fumarate alleviates allergic asthma by strengthening the Nrf2 signaling pathway in regulatory T cells
Source: Front Immunol. 2024 Apr 22;15:1375340. doi: 10.3389/fimmu.2024.1375340 (PMC11070462; doi:10.3389/fimmu.2024.1375340)
Supplement: Supplementary file 2 [file DataSheet_2.pdf]

Original blot images corresponding to the Fig. 5a in the manuscript.

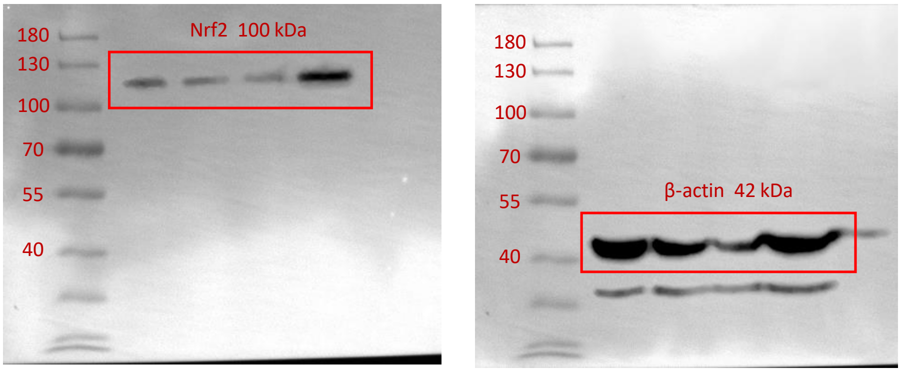

Original blot images corresponding to the Fig. 5e in the manuscript.

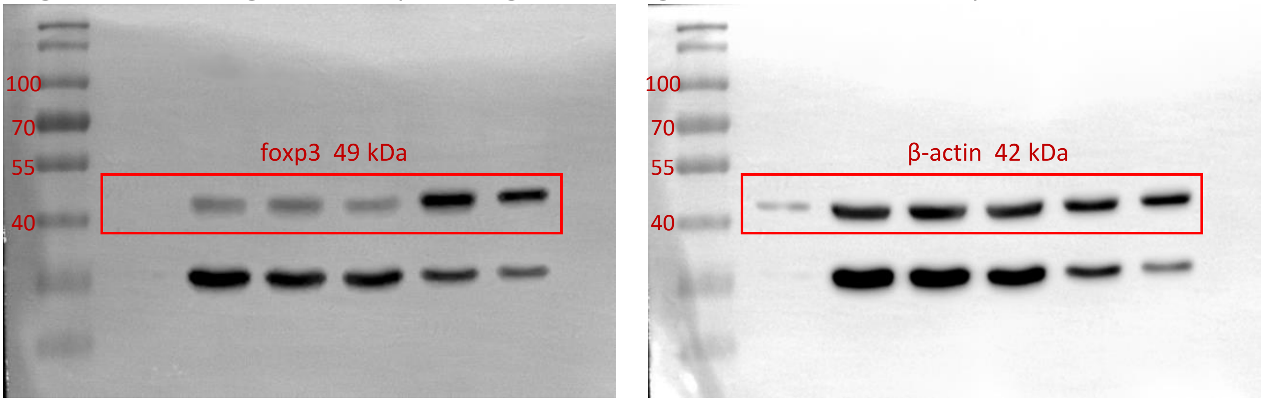

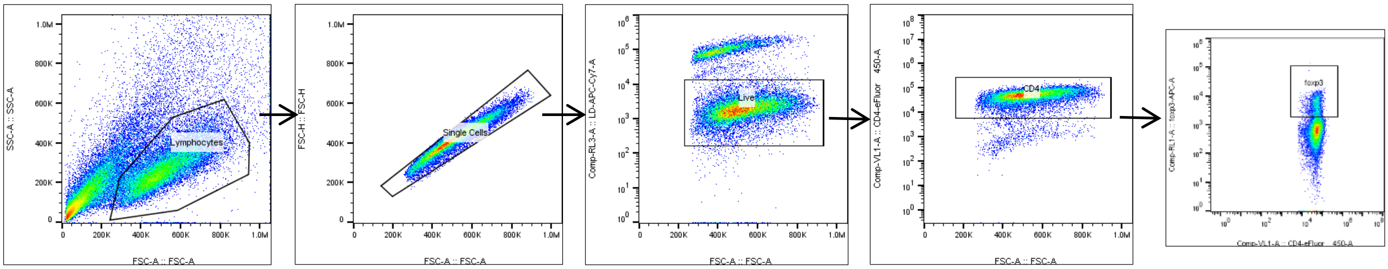

Gating strategy for flow cytometry analysis used in this study for Fig.5d and Fig.6d. Gating strategy used to define the population of foxp3^{+} T cells subsets from Naive CD4+ T cells after induction.
